# NOD-Like Receptors in Intestinal Homeostasis and Epithelial Tissue Repair

**DOI:** 10.3390/ijms15069594

**Published:** 2014-05-30

**Authors:** Marianna Parlato, Garabet Yeretssian

**Affiliations:** 1 Department of Medicine, Immunology Institute, Icahn School of Medicine at Mount Sinai, New York, NY 10029, USA; E-Mail: marianna.parlato@mssm.edu; 2 Tisch Cancer Institute, Icahn School of Medicine at Mount Sinai, New York, NY 10029, USA

**Keywords:** nucleotide-binding and oligomerization domain-like receptors (NLRs), intestinal epithelial cells (IECs), wound healing, colitis, inflammatory bowel diseases (IBD), inflammasome, growth factors, innate immunity, toll-like receptors (TLRs)

## Abstract

The intestinal epithelium constitutes a dynamic physical barrier segregating the luminal content from the underlying mucosal tissue. Following injury, the epithelial integrity is restored by rapid migration of intestinal epithelial cells (IECs) across the denuded area in a process known as wound healing. Hence, through a sequence of events involving restitution, proliferation and differentiation of IECs the gap is resealed and homeostasis reestablished. Relapsing damage followed by healing of the inflamed mucosa is a hallmark of several intestinal disorders including inflammatory bowel diseases (IBD). While several regulatory peptides, growth factors and cytokines stimulate restitution of the epithelial layer after injury, recent evidence in the field underscores the contribution of innate immunity in controlling this process. In particular, nucleotide-binding and oligomerization domain-like receptors (NLRs) play critical roles in sensing the commensal microbiota, maintaining homeostasis, and regulating intestinal inflammation. Here, we review the process of intestinal epithelial tissue repair and we specifically focus on the impact of NLR-mediated signaling mechanisms involved in governing epithelial wound healing during disease.

## 1. Introduction

Recurrent damage following injury of the intestinal epithelial barrier is at the basis of several intestinal disorders including inflammatory bowel disease (IBD), ischemic colitis and enteroinvasive bacterial infections. IBD, comprising the two clinically distinct forms, Crohn’s disease (CD) and ulcerative colitis (UC), is characterized by inappropriate and intermittent episodes of intestinal inflammation. IBD affects the health of more than 1.4 million people in North America and over 2.2 million individuals in Europe [1,2]. The incidence and prevalence of IBD continue to increase with time particularly in low frequency areas such as Asia, North Africa, Australia and New Zealand [3]. The pathophysiology and etiology of IBD are still unclear, however, it is thought to result from an exuberant inflammatory response to commensals and an impaired mucosal healing in genetically predisposed individuals [4,5]. The intestinal mucosa is colonized by tens of trillions of microbes that are either harmless or provide beneficial functions to the host [6]. This complex microbiota shapes the intestinal physiology predominantly by maintaining immune homeostasis [7], protecting against enteric pathogen colonization [8], and procuring nutrient metabolism [9]. It appears that tolerance mechanisms towards the commensal microbiota are dysregulated in IBD patients [10,11,12]. These patients show profound alterations in their gut microbiota composition, suggestive of an imbalance or “dysbiosis” between certain microbes that exacerbate inflammation and others that mitigate inflammation [13,14,15,16]. Sequencing of the 16S ribosomal DNA of the “normal” gut microbiota highlights the predominance of species from two bacterial divisions represented by the Bacteroidetes and the Firmicutes phyla [17,18]. Indeed, clinical dysbiosis was linked to CD with greater preponderance of certain Bacteroidetes and reduced complexity of the Firmicutes phylum [19,20]. Likewise, increased Proteobacteria and Actinobacteria and a concomitant depletion in Bacteroidetes was found in mucosal biopsies from UC patients compared to non-IBD controls [21,22]. Evidence obtained from animal models emphasizes the key role of certain commensals in dictating mucosal immunity by directly suppressing the inflammatory response or promoting the expansion of regulatory T (T_reg_) cells [23]. For instance, depletion of the mouse commensal segmented filamentous bacteria (SFB) leads to an impaired capacity of CD4^+^ T cells to differentiate into IL-17 producing T helper (T_H_) 17 subset, essential for host resistance to enteric infections [24,25,26]. Moreover, certain *Clostridium* strains promote Foxp3^+^ T_reg_ accumulation in a transforming-growth factor-β (TGF-β) rich milieu, indicating that indigenous species contribute to directly control systemic immune responses by inducing polarization of protective T cells [27].

While microbiota and environmental factors are essential contributors to the pathogenesis of IBD, genetic predispositions play a central role in IBD by disturbing the balanced and symbiotic/mutualistic host-microbiota interactions [28]. Recently, genetic studies have identified 163 IBD risk loci with 110 loci associated with both disease phenotypes, 30 specifically linked with CD and 23 with UC [29]. Interestingly, classified loci converge into common biological pathways essential for intestinal homeostasis- or inflammation-related processes, such as epithelial wound repair (e.g., *REL* [30], *STAT3* [31], *HNF4A* [32]) and barrier function (e.g., *ITLN1* [33], *MUC19* [31]), innate immune responses (e.g., *NOD2* [34], *CARD9* [35], *RIPK2* [29], *NLRP3* [36]), autophagy (e.g., *ATG16L1* [37], *IRGM* [38], *LRRK2* [39]), T-cell differentiation (*IL23R* [40,41]; *IL10R* [21,42], *JAK2* [43]), *etc*.

The intestinal mucosal surface is covered with epithelial cells constituting a physical barrier that prevents the entry of luminal antigens into the system [44,45]. Upon injury, the epithelial barrier undergoes a tightly regulated wound healing process involving various cytokines, growth factors, regulatory peptides, short chain fatty acids, bile acids, *etc*. [46]. Moreover, signaling cascades downstream of the innate immune Toll-like receptors (TLRs) and Nucleotide binding and oligomerization domain (NOD)-like receptors (NLRs) are also implicated in modulating epithelial barrier repair and integrity [47,48,49]. In this review, we will describe the mechanisms by which the intestinal epithelium is repaired following injury or during IBD. We will pay particular attention to the emerging role of NLRs in intestinal homeostasis and inflammation and discuss their potential in controlling signal transduction of intestinal tissue repair.

## 2. Intestinal Epithelial Barrier

The epithelium is a single layer of cells lining the inner surface of the gastrointestinal tract presenting different secretory and absorptive properties along the proximo-distal axis [50]. The small intestine consists of invaginations called crypts of Lieberkühn lined with finger-like protrusions or villi, whereas epithelial cells in the colon are regularly arranged in crypts. Four major cell lineages, namely columnar cells or enterocytes, goblet cells, enteroendocrine cells and Paneth cells, reside in the intestinal epithelium and display all the features of differentiated mature epithelial cells. Absorptive enterocytes are the most abundant population of polarized cells that not only regulate paracellular antigen trafficking via the tight junctions but also, in an undefined mechanism, process and present antigens directly to T cells [51,52,53]. Goblet cells and hormone-producing enteroendocrine cells are present in the entire intestinal epithelium of both crypts and villi [54]. Goblet cells secrete highly glycosylated gel-forming mucins (e.g., MUC2) that constitute the mucus, a paramount defense line of the gut epithelial barrier [54,55,56]. MUC2 is the major secreted intestinal mucin, however it acts differently throughout the proximo-distal axis of the intestine probably due to differential processing mechanisms [57]. The small intestine has a single layer of loose mucus, whereas the colon has a double-layered mucus, an outer loose and an inner attached mucus [54]. Increasing evidence underscores the importance of the concept that a defective mucus layer enhances the epithelial cell-microbiota interactions and therefore host susceptibility to intestinal injury. In fact, lack of MUC2 in mice results in severe colitis propagated by increased bacterial adhesion to the epithelium and intestinal permeability [58]. Paneth cells, the major source of antimicrobial peptides in the small intestine, are confined to the bottom of the crypts where they constitute a niche for multipotent leucine-rich repeat-containing G-protein coupled receptor 5 (Lgr5)-expressing intestinal stem cells (ISCs) [59].

During homeostatic turnover, epithelial cells are constantly expelled from the epithelial monolayer and die by anoikis, however barrier integrity is maintained [60]. Indeed, the intestinal epithelium undergoes rapid self-renewal every 4–5 days, and in order to retain optimal barrier functions and homeostatic cell numbers the epithelium employs a highly dynamic process involving crowding-induced extrusion of cells and their subsequent death [60,61,62]. The epithelial barrier is organized into three compartments along the crypt-to-villi axis: the base corresponds to the stem cell niche, the middle compartment comprises the transit-amplifying (TA) cells, and the upper part contains the differentiation zone, which expands until the surface epithelium of the crypts in the colon and the tip of the villi in the small intestine [63]. ISCs at the base of the crypts regularly divide to produce highly proliferative daughter TA cells that migrate upwards and become progressively committed to the absorptive or secretory cell lineages through activation of complex signal transduction cascades [64]. The Lgr5-expressing ISCs are virtually able to raise all cell types present in the epithelium [65,66,67], while another subset of ISCs known as +4 stem cells are quiescent and might serve as “reserve” stem cells [68]. Paneth cells have a slower turnover compared to other intestinal cell lineages; they arise from a small subset of dedicated Lgr5-expressing precursors and differentiate following a downward migratory path to the crypt bottom [69]. Animal models emphasize the long-term regenerative abilities of the intestinal epithelium during radiation- or chemically-induced injury providing key mechanistic insights into ISC-mediated tissue repair processes [70]. Several *ex vivo* culture systems and in particular the Matrigel-based three-dimensional culture system are built around the expansion of purified LGR5-expressing cells in a milieu enriched with growth factors, surrogate of the environment in the stem cell niche [59,71]. These *in vitro* expanded cells or epithelial organoids were shown to successfully repair and regenerate damaged colonic epithelia in mice [72]. Likewise, human and mouse immature fetal intestinal progenitors can be exploited to regenerate a differentiated region-specific epithelium, suggesting that stem cell-based therapies might be considered as potential treatment for IBD patients [73]. The lamina propria underneath the epithelial compartment consists of sub-epithelial connective tissue, non-hematopoietic mesenchymal or stromal cells and, among others, immune cells such as macrophages, dendritic cells, T and B lymphocytes [74].

## 3. Process of Intestinal Epithelial Tissue Repair

Impaired intestinal epithelium is a hallmark for various diseases and may result in the dissemination of toxic and immunogenic luminal substances into the system causing dysregulated gut homeostasis and extensive inflammation [75,76]. Hence, rapid sealing of the epithelium or epithelial wound healing after injury is necessary to reinstate this balance [77,78,79]. Complex and highly coordinated processes restore the continuity of the epithelial barrier in three distinct but overlapping steps: epithelial restitution, proliferation and differentiation [48,80]. Restitution begins within minutes to hours when epithelial cells lining the injured surface undergo de-differentiation, remodel the actin cytoskeleton and migrate over the wound to cover the damaged area [46]. Proliferation starts hours or days after the injury to increase the pool of enterocytes and is followed by the re-differentiation step mainly to reestablish barrier integrity and function. During epithelial restitution, actin reorganization provides a structural framework that defines the shape and polarity of the epithelial cells and that creates traction forces necessary to push the cell forward [81]. This dynamic mechanism is tightly controlled by the RHO family of small guanine triphosphatases (GTPases; e.g., RHO, RAC and CDC42) [82]. Principally, CDC42 and RAC are respectively required for the development of filopodia and lamellipodia protrusions that facilitate the transient adherence of the epithelial cells to the underlying matrix [83], whereas RHO regulates the polymerization of actin to produce stress fibers [84]. Crawling of intestinal epithelial cells (IECs) during restitution can be enhanced *via* chemokine activation. In fact, RHO-GTPase activation enhances CXCL12-CXCR4-mediated actin rearrangement promoting epithelial cell migration and barrier restitution [85]. Similarly, CCL20 and the human β-defensin 2 (HBD2) through engagement with CCR6 direct actin accumulation and IEC migration [86]. Certain intestinal epithelial molecules such as Villin, an actin-binding protein expressed predominantly in the villi, also participate in tissue repair by apical pole remodeling of migrating cells [87,88]. Villin localizes at the lamellipodia favoring cellular motility *in vitro* and *in vivo* through calcium-dependent wrapping, capping, and severing of actin filaments [89,90,91,92]. Low levels of Villin expression are observed in IECs derived from IBD patients, and Villin-deficient mice are more susceptible to dextran sulfate sodium (DSS) [93,94,95], a sulfated polysaccharide highly toxic to crypt enterocytes and commonly used for experimental acute injury and intestinal tissue repair [96,97].

## 4. Mechanisms of Intestinal Epithelial Tissue Repair

**Figure 1:**
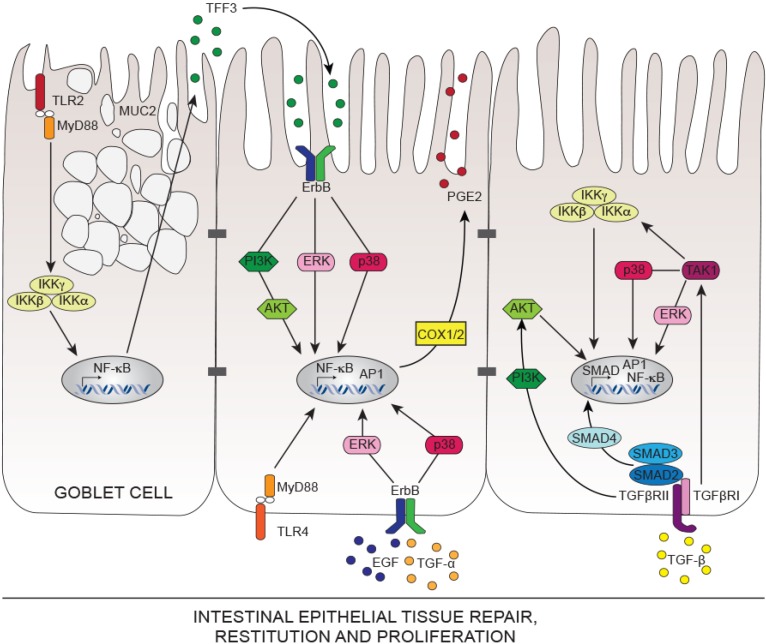
Signal transduction pathways involved in intestinal epithelial tissue repair. TLR2 engagement induces TFF3 release from mucin (MUC2) secreting Goblet cells. Released TFF3 is detected by adjacent enterocytes via EGFR (ErbB) transactivation leading to PI3K and MAPK (ERK, p38) signaling. Similarly, TLR4 activation at the basal side of the IECs permits COX1/2-derived PGE2 secretion essential for efficient cell proliferation. Both EGF and TGF-α induce epithelial restitution and proliferation by stimulating ErbB receptors and activating downstream p38, ERK and NF-κB signaling cascades. On the other hand, TGF-β via its type I and II serine/threonine receptors induces the translocation of SMAD2/3/4 complex to the nucleus and regulates a gene transcription program fundamental for epithelial cell differentiation and restitution.

Multiple regulatory growth factors, immunoregulatory cytokines and peptides control intestinal wound healing by either impacting the restitution or the proliferation processes. Many of these factors promote tissue repair by enhanced production of bioactive TGF-β [98], however some other factors such as trefoil factors (TFF) and galectins promote wound healing in a TGF-β-independent mechanism [99,100]. Galectins are a family of β-galactoside binding lectins of which Galectins-2, -3 and -4 are expressed in IECs supporting cell migration and wound re-epithelialization [100,101]. Recent discoveries show that Galectin-3 levels are impaired in IBD patients and propose that Matrix metalloproteinase-7 (MMP7) cleaves Galectin-3 during intestinal inflammation hampering its tissue repair functions [102]. TFF, small protease-resistant peptides secreted by Goblet cells, play critical roles in intestinal homeostasis through potentiating epithelial restitution [99,103,104]. For instance, TFF3 deficiency renders mice highly susceptible to DSS-induced colitis due to an impaired mucosal protection [105]. Moreover, administration of human hTFF2 and transgenic overexpression of hTFF1 accelerate mucosal healing and decrease inflammation during experimental models of colitis [106,107,108,109]. So far no specific TFF receptor was described, however *in vitro* evidence put forward the idea that TFF3 induces the transactivation of ErbB family members of receptor tyrosine kinases, including epidermal growth factor receptor (EGFR/ErbB1) and ErbB2, and enhances IEC survival and proliferation in a p38 mitogen activated protein kinase (MAPK)- as well as phosphoinositol 3-kinase (PI3K)-dependent manner [110,111,112] (Figure 1). Additionally, tumor necrosis factor (TNF)-α and nuclear factor-kappa B (NF-κB) repress the transcription of intestinal TFF3 *in vitro*; suggesting that perpetual activation of NF-κB in the intestinal mucosa of IBD patients may promote ulcerations and hence decrease TFF3-dependent tissue repair [113]. It appears that NF-κB, master regulator of inflammation [114], has tissue-protective functions in the intestine as IEC-specific inhibition of NF-κB in mice through deletion of NEMO (also called IKKγ; IκB kinase-γ) [115], or both IKKα and IKKβ subunits spontaneously cause severe chronic intestinal inflammation [116,117,118].

Apart from TFF and Galectins, tissue repair factors expressed within the intestinal mucosa such as EGF, TGF-α, TGF-β, hepatocyte growth factor (HGF), granulocyte macrophage colony stimulating factor (GM-CSF), vascular endothelial cell growth factor (VEGF), keratinocyte growth factor (KGF), platelet-derived growth factor (PDGF), as well as interleukin (IL)-1β, IL-2 and interferon (IFN)-γ cytokines activate specific signaling pathways that converge to induce bioactive TGF-β-mediated epithelial cell restitution [46,98,119,120,121,122,123,124,125,126]. Most of these regulatory molecules reduce mucosal damage and intestinal inflammation in *in vivo* models of colitis [127,128,129,130,131], however EGF family peptides (EGF, TGF-α and TGF-β) occupy a central role in the healing mechanisms and therefore will be discussed more extensively. In the intestine, EGF and TGF-α control IEC proliferation and regeneration. Both factors are necessary for healing of the injured intestinal mucosa where the majority of cells express EGFR, the common receptor for EGF and TGF-α. Interestingly, mucosal inflammation enhances EGFR expression, yet IBD patients have impaired TGF-α expression and low serum EGF levels [132,133,134]. Transactivation of EGFR triggers MAPK p38 and ERK (extracellular signal regulated-kinases) signaling, which is critical for mucosal defense and for protecting IECs from TNF-α-mediated apoptosis [135,136,137] (Figure 1). Mice lacking TGF-α are highly susceptible to DSS or trinitrobenzene sulphonic acid (TNBS) induced colitis, a phenotype that is reversible by systemic administration of TGF-α [138]. Similarly, EGF largely contributes to restore the epithelial barrier integrity after chemical injury [139]. Alongside, pharmacological inhibition of MAPK signaling aggravates DSS- and TNBS-induced colitis in mice [140].

Although TGF-β plays a crucial role in reestablishing homeostasis and tissue repair after injury, it inhibits IEC proliferation [141]. TGF-β deficiency in mice mediates spontaneous intestinal inflammation [142], extending earlier discoveries that compromised TGF-β signaling contributes to the development of IBD through upregulation of the negative regulator Smad-7 [143,144]. TGF-β signals through type I and type II serine/threonine receptors and sequentially triggers the activation of Smad-2, -3, and -4 transcription cofactors [145]. Lack of TGF-β signaling in transgenic mice expressing a dominant negative functionally inactive form of TGFβRII on epithelial cells increases susceptibility to intestinal injury and delays wound healing *in vivo* [146]. Various studies indicate the presence of Smad-independent TGF-β signal transduction pathways critical for intestinal tissue repair [147,148,149]. In fact, TGF-β rapidly activates PI3K/AKT, MAPK p38 and ERK and TGF-β activated kinase 1 (TAK1) pathways to induce wound healing. For example, IEC-specific TAK1 signaling prevents TNF-dependent apoptosis and its absence disrupts intestinal homeostasis favoring inflammation [150,151,152] (Figure 1).

While most intestinal tissue repair mechanisms involve the epithelial factors described above, deeper lesions require non-epithelial cell populations and other inflammatory processes. Interestingly, stromal cells underlying the epithelial barrier are in perpetual crosstalk with IECs during homeostasis and repair after injury [153]. Recent studies show that Wnt5a-expressing stromal cells are key for intestinal tissue regeneration [154,155]. Secretion of the non-canonical ligand Wnt5a amplifies TGF-β signaling, which in turn represses proliferation of adjacent IECs allowing these later to arrange into new crypt units [155,156]. Stromal cells as well as IECs are a major source of Prostaglandin-E2 (PGE2) that prevents diminished epithelial proliferation accompanying radiation- or DSS-mediated injury in mice [157,158]. PGE2 is synthesized through activation of cyclooxygenase-1 (COX-1) or COX-2 reflected by the fact that mice deficient of either of these enzymes show decreased epithelial proliferation following DSS administration [159,160,161,162] (Figure 1). Interestingly, PGE2 producing-stromal cells relocate from the lamina propria to the bottom of the crypts during tissue injury and trigger mechanisms to actively support the intestinal epithelial restitution and tissue repair [74,161].

## 5. Innate Immunity Controlling Intestinal Tissue Repair

Commensal microbiota extensively contributes to intestinal homeostasis through a wide range of mechanisms specifically involving epithelial wound healing. Early studies put forward the idea that mice bred in germ-free conditions or wild-type mice treated with broad-spectrum antibiotics are highly susceptible to DSS-induced injury, suggesting that commensal microbiota sensing is required for optimal epithelial cell restitution and proliferation [163,164]. Interestingly however, seminal studies support the concept that microbiota is required for the initiation of intestinal inflammation with an important implication of innate immune recognition receptors in the development of commensal-dependent colitis [165,166,167].

### 5.1. Toll-Like Receptors TLRs in Intestinal Tissue Repair

Toll-like receptor (TLR) expression and activation is specifically regulated in the intestine to contrive continuous reaction to commensal derived molecular patterns. In the meantime, intestinal epithelial TLRs retain the ability to sense and combat invading pathogens [168]. Surface expression of TLRs is decreased in IECs and is relocated to intracellular compartments or to the basolateral surface of the cell [169,170,171,172,173,174,175]. Numerous *in vivo* and *in vitro* studies highlight the contribution of TLR signaling to intestinal tissue repair after injury [168,176]. In fact, mice deficient in the shared TLR signaling adaptor protein Myeloid Differentiation Primary Response 88 (MyD88) show severe intestinal inflammation and increased susceptibility to experimental or infectious colitis, due to impaired epithelial restitution and intestinal tissue repair [163,177,178,179,180,181]. Similarly, mice lacking TLR2, TLR4, TLR5 or TLR9 present unbalanced microbial sensing that provokes epithelial barrier injury and exacerbated intestinal inflammation during colitis [182,183,184,185,186]. Engaging the TLR signaling preserves intestinal epithelial barrier integrity by enhancing transepithelial resistance of IECs [187], by protecting intercellular tight junctions [188] or by promoting the expression of various proliferative and anti-apoptotic factors [151,189,190,191]. Indeed, TLR4 signaling is required to induce COX-2 activation, which leads to PGE2 production by IECs and subsequent induction of an EGF family growth factor, amphiregulin [192] (Figure 1). Moreover, TLR signaling within IECs confers protection in acute DSS-induced colitis by increasing the expression of amphiregulin in a MyD88 and TIR-domain-containing adapter-inducing interferon-β (TRIF) dependent manner [193]. Following intestinal injury, TLR9 upregulates VEGF and an intestinal progenitor cell differentiation factor hairy enhancer of split 1 (HES1) to potently drive epithelial tissue repair [194]. Also, TLR2 activation induces secretion of TFF3 by Goblet cells, which once sensed by the IECs fosters their restitution and proliferation [191,195]. Besides, during intestinal inflammation stromal cells and myeloid cells are recruited to the intestinal stem cell niche, where they promote proliferation and survival of epithelial progenitors through secretion of a myriad of regulatory factors that render the milieu highly restitutive and proliferative [161,196].

### 5.2. NOD-Like Receptors (NLRs) in Intestinal Homeostasis, Inflammation and Tissue Repair

The human NOD-like receptor (NLR) family of intracellular sensors comprises 22 members, which share a common modular tripartite organization: *C*-terminal leucine-rich repeats (LRR) implicated in ligand sensing, central nucleotide-binding and oligomerization domain (NOD also called NACHT) and *N*-terminal protein-protein–interaction domain. This later prompts NLR activity and their classification into four subfamilies: NLRA containing an acidic domain, NLRB with a baculovirus inhibitor of apoptosis repeat (BIR) domain, NLRC consisting of NLRs with a caspase activation and recruitment domain (CARD), and NLRP with a pyrin domain (PYD) [197]. NLRX1 is an exception, as it comprises a non-identified CARD-related X effector domain [198,199,200]. Upon sensing a wide array of microbial- or danger-associated molecular patterns, MAMPs and DAMPs respectively, the NLRs undergo conformational changes with the LRR domain, exposing the *N*-terminal domain and allowing protein-protein interactions with downstream signaling adaptor molecules. The culmination of these interactions drives the expression of inflammatory and antimicrobial genes that govern both innate and adaptive immune responses.

#### 5.2.1. Non-Inflammasome NLRs

**Figure 2:**
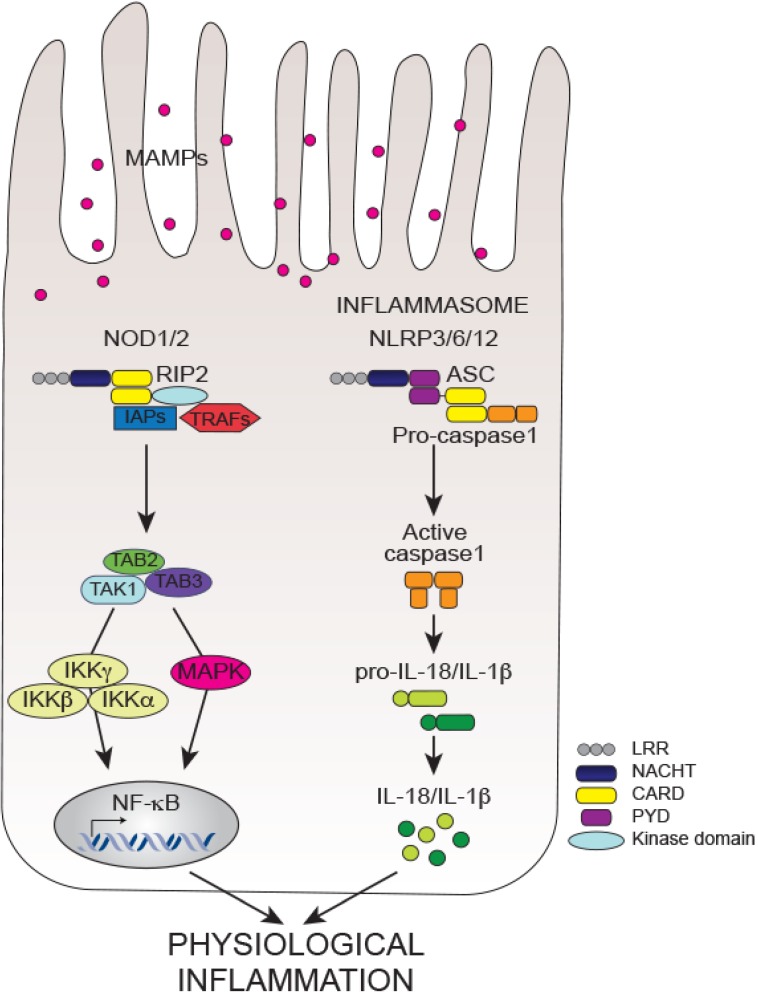
Simplified NLR pathways engaged in intestinal homeostasis. Upon microbial sensing, NOD1 and NOD2 recruit the adaptor protein RIP2 via CARD-CARD interactions. Activated RIP2 binds to the E3 ubiquitin ligases TRAFs and IAPs triggering NF-κB and MAPK signaling. Similarly, the adaptor protein ASC bridges the activated inflammasome NLRs (NLRP3, 6 and 12) to pro-caspase-1 through both PYD and CARD domains, respectively. Formation of the inflammasome complex triggers auto-proteolytic cleavage and activation of caspase-1 subsequently leading to maturation and release of IL-1β and IL-18. Activation of both NOD signaling and inflammasome pathways contributes to the maintenance of intestinal homeostasis against resident commensal microbes.

NOD1 (CARD4) and NOD2 (CARD15) are the first NLRs identified and play a major role in triggering innate immune signaling following bacterial sensing. NOD1 and NOD2 discriminate between Gram-negative and Gram-positive bacteria by sensing specific peptidoglycan motifs. For instance, NOD2 detects the common bacterial muramyl-dipeptide (MDP), while NOD1 recognizes a subset of bacteria that contain the d-glutamyl-meso-diaminopimelic acid (DAP) found in Gram-negative bacteria but also in some Gram-positive bacteria like *Listeria monocytogenes* and *Bacillus* species [201,202,203]. Upon ligand sensing, NOD1 and NOD2 via homotypic CARD–CARD interactions recruit the adaptor kinase receptor-interacting protein 2 (RIP2, also known as RIPK2) and form large macromolecular complexes [204,205,206]. Lack of RIP2 in mice abrogates ligand-mediated NOD1 and NOD2 signaling, emphasizing the central role that RIP2 plays in this cascade [205,207]. RIP2 activation occurs via Lysine (K)63-polyubiquitination mediated by several E3 ubiquitin ligases, including X-linked inhibitor of apoptosis (XIAP) [208], cellular inhibitor of apoptosis protein 1 (cIAP1) and cIAP2 [209], TNFR-associated factor 2 (TRAF2), TRAF5 and TRAF6 [210,211]. Polyubiquitination of RIP2 is accompanied with its autophosphorylation on tyrosine (Tyr474), which promotes NF-κB signaling [212]. RIP2 then directly binds to the TAK-1/TAB-2/TAB-3 (TAK-1-binding protein 2 and 3) complex and recruits IKK complex, which in turn leads to downstream activation of both NF-κB and MAPK pathways [204,213,214] (Figure 2). Recent genome-wide RNAi screens put forward novel regulators of the NOD1/2-NF-kB signaling axis [215,216,217]. For instance, NOD1 and NOD2 engage in a crosstalk with apoptosis proteins through an interaction with the pro-apoptotic protein BH3-interacting domain death agonist (BID) [215,218]. Notably, loss of BID impairs NOD1- and NOD2-mediated innate immunity by differentially regulating NF-κB and ERK signaling [215]. Activation of NOD1 and NOD2 results in the production of an array of antimicrobial peptides (e.g., defensins [45]) as well as pro-inflammatory cytokines (e.g., TNF-α, IL-6, *etc*.) and chemokines (e.g., CC-chemokine ligand 2 (CCL2), CXC-chemokine ligand 8 (CXCL8 or IL-8), CXCL2, *etc*.) [49,219,220].

While structurally similar, NOD1 and NOD2 diverge in their expression profiles as NOD1 is found in a wide variety of cell types whereas the expression of NOD2 is confined to myeloid and lymphoid cells [221]. However, IECs as well as small intestinal Paneth cells express NOD2 [201,222]. Consistently, increased levels of NOD2 are observed in Paneth cells from IBD patients [223]. In fact, treatment of colonic IECs with TNF-α, IFNγ or butyrate regulate the synthesis of NOD2 at the mRNA level, likely indicating that inflammation during IBD drives the increase in NOD2 signaling [224,225,226]. *NOD2* is the first identified gene associated with CD susceptibility and until now the strongest genetic risk factor for the development of CD [29,34,227]. In particular, three coding variants within *NOD2* (p.G908R, p.R702W and p.L1007fs) possess the greatest risk associated with CD so far [34,227,228]. All three variants fall within the LRR region, and therefore result in decreased MDP responsiveness [229]. Unlike *NOD2*, genetic studies linking polymorphisms in *NOD1* with IBD susceptibility are still conflicting and necessitate further investigation [230,231]. Given the implication of NOD1 and NOD2 in bacterial sensing, their expression in IECs and the strong association of *NOD2* with CD, it is speculated that altered detection of commensals might impair intestinal homeostasis and contribute to intestinal inflammation. Evidence from *in vitro* and *in vivo* experiments support this concept and underscore the importance of the cellular localization of NOD proteins and their antimicrobial functions (reviewed in [45]) in the intestine. During infections with enteric pathogens, cytosolic NOD1 and NOD2 interact with the autophagy-related protein 16-like 1 (ATG16L1) and are recruited to the plasma membrane. Also, FERM and PDZ domain protein-containing 2 (FRMPD2) cooperate with NOD2 at the basolateral membrane of IECs providing a spatial specificity to MDP-mediated NOD2 signaling [216]. Concomitantly, FRMPD2 links NOD2 to the membrane-associated ERBB2-interacting protein ERBIN [216,232,233]. Small RHO GTPases are well-described targets of bacterial virulence factors but also critical for epithelial cell migration and barrier restitution after injury [85,234]. Recent data shows that RHO GTPases form a complex with NOD1 and NOD2, further promoting their assembly at the plasma membrane and suggesting that cytoskeleton remodeling regulates NOD signaling [235,236,237]. Notably, activation of RHOA, RAC1 and CDC42 prompt NOD1 signaling in both ligand dependent- and independent-manner [236].

Murine models are invaluable tools to unravel the mechanisms governing NOD1- and NOD2-mediated intestinal homeostasis and inflammation. For instance, administration of peptidoglycan or MDP negatively regulates TLR signaling and protects wild type mice from colitis, suggesting that the microbial sensing by NOD proteins at the gut immune barrier is key for host protection [238,239]. Furthermore, mice deficient of NOD1, NOD2 or double knockout mice for NOD1 and NOD2 are extremely susceptible to chemically-induced colitis due to increased barrier permeability and lack of efficient epithelial tissue repair [240,241,242,243,244]. Given its expression in Paneth cells, NOD2 controls the production of antimicrobial peptides and thus preserves the balance between the gut commensal communities and confers protection during intestinal injury [245]. Similar to CD patients, NOD2-deficient mice present dysbiosis with reduced ability to prevent intestinal colonization of pathogenic bacteria and enhanced sensitivity to colitis [244,246,247]. Interestingly, when wild type mice are cohoused with NOD2-deficient mice and subjected to DSS-induced injury, they develop severe intestinal inflammation and epithelial barrier injury, a phenotype reversed by systemic neutralization of the pro-inflammatory cytokine IL-6 [244]. Akin to NOD2, NOD1 controls the composition of the intestinal commensal flora and is required for optimal generation of intestinal lymphoid tissue by directly detecting microbial components [248]. Along the same line of investigation, mice deficient in NOD2 or both NOD1 and NOD2 have a delayed response to Gram-negative *Citrobacter rodentium*, a rodent surrogate of human enteropathogenic *Escherichia coli* infection [219,249]. Indeed, NOD1 and NOD2 double deficient mice present an impaired capacity to mount appropriate T_H_17-type immune responses against *C. rodentium* infection [249]. On the other hand, NOD2 deficiency renders mice more susceptible to *C. rodentium* due to reduced CCL2 chemokine production, decreased chemotaxis of inflammatory monocytes to the intestine and subsequent compromised T_H_1 immune responses [219].

#### 5.2.2. Inflammasome NLRs

Apart from NOD1 and NOD2, other NLRs mediate the activation of the inflammatory caspases-1 and -11 in macromolecular platforms called inflammasomes subsequently promoting the secretion of the pro-inflammatory cytokines IL-1β and IL-18 and pyroptosis, a form of cell death induced by bacterial pathogens [250,251,252] (Figure 2). Inflammasome activation requires two signals: signal 1 is provided by TLR stimulation and NF-κB-dependent synthesis of the pro-forms of IL-1β and IL18; whereas signal 2 specifically induced by MAMPs or DAMPs promotes caspase-1 activation and processing of the pro-forms of IL-1β and IL-18 into their active mature forms. The inflammasome NLRs share the same tripartite architecture as all NLRs with either an *N*-terminal PYD or CARD domain, which allows homotypic interactions with the PYD domain of the adaptor protein apoptosis-associated speck-like protein (ASC; also called PYCARD) or the CARD domain of caspase-1, respectively [253,254,255]. ASC also contains a CARD domain that upon inflammasome activation with microbial- or danger-associated molecular patterns (MAMPs or DAMPs) binds to the CARD of caspase-1 [256]. Most inflammasomes play a central role in infectious, autoimmune and inflammatory diseases, however only few regulate intestinal homeostasis and inflammation including NLRP3, NLRC4, NLRP6 and NLRP12 [49,257].

Observations made using genetic studies emphasize the role of inflammasomes in the pathogenesis of IBD. Interestingly, polymorphisms in genes encoding IL-18 and IL-18 receptor accessory protein (IL-18RAP) are associated with increased CD pathology [258,259]. Consistently, polymorphisms in the regulatory region downstream of the human *NLRP3* gene are also linked to CD susceptibility, due for instance to decreased NLRP3 mRNA expression and impaired IL-1β production by lipopolysaccharide-challenged monocytes [36]. Recent evidence from murine models of experimental colitis support the genetic linkage analysis and accentuate the central role played by inflammasomes in the intestine [29,36]. Initial studies show that pharmacological inhibition of caspase-1 with Pralnacasan or neutralization of IL-18 confers resistance to experimental colitis [260,261]. Similarly, caspase-1-deficient mice on the Balb/c background have reduced disease after DSS-injury [262]. Interestingly however, recent discoveries refuted these earlier findings by purporting the importance of inflammasome signaling in maintaining tissue homeostasis and protection during injury [263,264,265,266]. Mice deficient in the inflammasome components ASC and caspase-1 are susceptible to DSS colitis and display heightened intestinal permeability and commensal bacterial translocation probably due to an impaired epithelial restitution and proliferation [263]. These mice exhibit a hampered IL-18 production by IECs upon acute intestinal injury, a phenotype rescued by exogenous administration of recombinant IL-18, further underscoring the requirement of IL-18 to efficiently repair and regenerate the epithelial barrier upon injury [263]. Equally, lack of epithelial IL-18 expression in NLRP3 deficient mice enhanced susceptibility of these mice to DSS-induced colitis when compared to control wild-type mice [265,267,268]. Notably, increased intestinal inflammation in NLRP3 knockout mice is associated with reduced levels of IL-1β, anti-inflammatory IL-10, and the IEC restitution factor TGF-β [268]. While most studies outlined above underscore a protective role for NLRP3 during intestinal inflammation, recent studies contradict these observations by showing that NLRP3-deficient mice are resistant to colitis [269,270]. In an infectious model of colitis with *C. rodentium*, early activation of the NLRP3 or NLRC4 inflammasomes within the IECs limit pathogen colonization and prevent pathology in mice [271,272]. These findings were confirmed in mice deficient of various inflammasome components including NLRP3, NLRC4, ASC, Caspase-1, IL-1β and IL-18 [271,272,273]. IL-18 receptor (IL-18R) signals through the common adaptor MyD88, which suggests that the IL-18/IL-18R/MyD88 signaling might be implicated in COX-1- and COX-2-mediated secretion of PGE2 essential for intestinal epithelial restitution and proliferation [160,161,189]. Consistently, recent reports further emphasize that IL1R, IL18R or IL18 deficiency renders mice highly susceptible to DSS-induced colitis indicating that signaling through MyD88 is required to preserve the intestinal barrier and to promote repair following injury [266,274,275]. Hence, IL-18 exerts both protective (in IECs) and harmful (in the lamina propria) effects in intestinal inflammation depending on the site of activation and the level of IL-18 produced [263,264,276]. Unlike other inflammasomes, NLRC4 activates caspase-1 and mediates IL-1β and IL-18 production in an ASC-dependent or independent manner [255,277]. The NLRC4 inflammasome is important in host defense against Gram-negative bacteria and its activation involves sensing of flagellin and components of the bacterial Type III secretion system [255,278,279]. Although loss of NLRC4 does not alter basal intestinal homeostasis, DSS-induced epithelial injury in mice lacking NLRC4 results in low expression of IL-1β and IL-18 and consequently severe disease [280]. However, another study using the same chemically induced colitis model shows opposite results, demonstrating an ameliorated severity of colitis in NLRC4-deficient mice [270]. These discrepancies may stem from the length and concentration of DSS used as well as differences in the composition of the intestinal microflora between the two facilities.

In addition, two recently described NLRP6 and NLRP12 inflammasomes also play a key role in the maintenance of gut homeostasis [281,282,283,284,285]. Both NLRP6 and NLRP12 cooperate with ASC to promote caspase-1 activation and production of IL-1β and IL-18 [286,287,288]. Moreover, *in vitro* studies suggest that both receptors potentiate NF-κB and MAPK signaling and require ASC to promote these responses [286,287]. In marked contrast, later findings show that NLRP6 is a negative regulator of canonical NF-κB signaling and that mice deficient in NLRP6 survive systemic bacterial infections through upregulation of NF-κB-mediated cytokines and enhanced bacterial clearance [289]. Moreover, NLRP12 negatively regulates the noncanonical NF-κB pathway by directly associating with the NF-κB inducing kinase (NIK) or IRAK-1 and interfering with TLR-mediated signaling [284,290,291]. NLRP6 deficient mice develop sever colitis in response to DSS when compared to wild type mice, manifested with reduced IL-18 expression by IECs and increased pathology [281,282,283]. The phenotype of NLRP6 mice is transmissible when cohoused together with wild type mice for 4 weeks, highlighting the colitogenic origin of the microbiota of NLRP6 mice. Notably, NLRP6 mice have an altered microbiota with expansion of pathobiont bacteria belonging to the genus *Prevotellaceae* as well as the phylum TM7 and present increased CCL5 production [281]. Broad-spectrum antibiotic treatment of these mice depletes both *Prevotellaceae* and TM7 and protects the mice from DSS-induced injury. In addition, another possible mechanism for NLRP6 mediated protection might be that NLRP6 limits epithelial cell depolarization and tissue disintegration upon injury by regulating downstream target genes of both Wnt and Notch signaling cascades [282]. Akin to NLRP6, loss of NLRP12 in mice causes more severe colitis, in line with the role of NLRP12 as negative regulator of NF-κB and MAPK pathways [284,285]. Interestingly however, both studies describing the colitic phenotype of the NLRP12-deficient mice propose opposing mechanisms using bone marrow chimera experiments without further characterization whether NLRP12 exerts its protective functions from the hematopoietic or non-hematopoietic compartments [257,284,285]. While lack of NLRs affects the composition of the commensal flora, little is know whether the protective functions of NLRs in the context of intestinal injury are direct. A recent report by Wlodarska *et al.* emphasizes that the NLRP6 inflammasome is a critical orchestrator of goblet cell mucin exocytosis and lack of NLRP6 leads to defective autophagy and mucin secretion, two key regulators implicated in maintaining intestinal homeostasis and protection during colitis [292]. This suggests that NLRs in general and NLRP6 in particular might indirectly protect the host from intestinal commensals during injury by employing the host’s physiological defense mechanisms such as the autophagy machinery.

## 6. Conclusions

Exciting progress has been made in understanding the signal transduction mechanisms governing mucosal wound healing after physical, chemical or bacterial injury. Based on the above discussions, intestinal tissue repair arises as a highly orchestrated and finely tuned process involving, not only regulatory peptides, growth factors and cytokines, but also innate immune recognition. A large body of recent research emphasizes the significant role of host innate immune NLRs in promoting intestinal health. NLRs maintain intestinal mucosal integrity and homeostasis by controlling tolerance to the commensal microbiota, by regulating inflammatory signaling events, and by amplifying the crosstalk between IECs and underlying hematopoietic and non-hematopoietic cells. Genetic studies correlating loss of function mutations in the *NOD2* gene or in the regulatory region of the *NLRP3* gene with CD susceptibility further accentuate the prominence of NLRs in tissue repair processes at the gut immune barrier. Emerging experimental and clinical evidence suggests that NLR-mediated signals protect the host from intestinal inflammation endorsing the concept that these innate immune sensors prompt IEC restitution and elicit reestablishment of homeostasis. However, various mechanisms may be involved in driving mucosal healing by NLRs. Hence, additional studies are required to definitively clarify the relative contributions of the different NLR-mediated signal transduction mechanisms in the regulation of intestinal homeostasis and disease. The full understanding of these cascades of events may set the stage for novel therapeutic strategies targeting epithelial tissue repair in IBD patients by specifically modulating NLR functions within the IECs.

## Abbreviations

IECs: intestinal epithelial cells
IBD: inflammatory bowel diseases
NLRs: nucleotide-binding and oligomerization domain-like receptors
TLRs: toll-like receptors
CD: Crohn’s disease
UC: ulcerative colitis
T_H_: T helper
TGF-β: transforming-growth factor-β
Foxp3: forkhead box P3
*STAT3*: signal transducer and activator of transcription 3
*HNF4A*: hepatocyte nuclear factor 4 alpha
*ITLN1*: intelectin 1
*MUC19*: mucin 19
*NOD2*: nucleotide-binding oligomerization domain containing 2
*CARD9*: caspase recruitment domain family, member 9
*RIPK2*: receptor interacting protein kinase 2
*NLRP3*: NLR family, pyrin domain containing 3
*ATG16L1*: autophagy related 16-like 1
*IRGM*: immunity-related GTPase family, M
*LRRK2*: leucine-rich repeat kinase 2
*IL23R*: interleukin 23 receptor
*IL10R*: interleukin 10 receptor
*JAK2*: janus kinase 2
MUC2: mucin 2
Lgr5: leucine-rich repeat-containing G-protein coupled receptor 5
TA: transit-amplifying
ISCs: intestinal stem cells
GTPases: guanine triphosphatases
RHO: rhodopsin
CDC42: cell division cycle 42
CXCL: chemokine (C-X-C motif) ligand
HBD2: human β-defensin 2
CCL2: chemokine (C-C motif) ligand 2
CCR6: chemokine (C-C motif) receptor 6
DSS: dextran sulfate sodium
MMP7: Matrix metalloproteinase-7
TFF: trefoil factor
EGFR: epidermal growth factor receptor
EGF: epidermal growth factor
ErbB: v-erb-b2 avian erythroblastic leukemia viral oncogene homolog
MAPK: mitogen activated protein kinase
PI3K: phosphoinositol 3-kinase
TNF-α: tumor necrosis factor alpha
NF-κB: nuclear factor-kappa B
ΙΚΚα: IκB kinase alpha
ΙΚΚβ: IκB kinase beta
ΙΚΚγ: IκB kinase gamma
NEMO: NF-κB Essential Modulator
HGF: hepatocyte growth factor
VEGF: vascular endothelial cell growth factor
GM-CSF: granulocyte macrophage colony stimulating factor
KGF: keratinocyte growth factor
PDGF: platelet-derived growth factor
IFN: interferon
ERK: extracellular signal regulated-kinases
TNBS: trinitrobenzene sulphonic acid
COX1/2: cyclooxygenase 1/2
SMAD: Sma- And Mad-Related Protein
TGFβRII: TGFβ receptor II
TAK1: TGF-β activated kinase 1
Wnt5a: wingless-type MMTV integration site family, member 5A
PGE2: Prostaglandin-E2
MyD88: Myeloid Differentiation Primary Response 88
TRIF: TIR-domain-containing adapter-inducing interferon-β
HES1: hairy enhancer of split 1
LRR: leucine-rich repeats
NACHT: nucleotide-binding and oligomerization domain
BIR: baculovirus inhibitor of apoptosis repeat
CARD: caspase activation and recruitment domain
PYD: pyrin domain
MAMPs: microbial-associated molecular patterns
DAMPs: danger-associated molecular patterns
DAP: d-glutamyl-meso-diaminopimelic acid
MDP: muramyl dipeptide
XIAP: X-linked inhibitor of apoptosis
cIAP: cellular inhibitor of apoptosis protein
TRAF: TNFR-associated factor
TAB2-TAB3: TAK-1-binding protein 2 and 3
RNAi: ribonucleic acid interference
FRMPD2: FERM and PDZ domain protein-containing 2
ERBIN: ERBB2-interacting protein
ASC: apoptosis-associated speck-like protein
IL18RAP: Interleukin 18 Receptor Accessory Protein
